# Persistent foci of falciparum malaria among tribes over two decades in Koraput district of Odisha State, India

**DOI:** 10.1186/1475-2875-12-72

**Published:** 2013-02-21

**Authors:** Sudhansu Sekhar Sahu, Kasinathan Gunasekaran, Perumal Vanamail, Purusothaman Jambulingam

**Affiliations:** 1 Vector Control Research Centre (ICMR), Medical Complex, Indira Nagar, Puducherry, 605006, India

**Keywords:** *Plasmodium falciparum*, Koraput, Odisha, India

## Abstract

**Background:**

Koraput, a predominantly tribe-inhabited and one of the highly endemic districts of Odisha State that contributes a substantial number of malaria cases to the India’s total. Control of malaria in such districts would contribute to change the national scenario on malaria situation. Hence, a study was carried out to measure the magnitude of malaria prevalence in the district to strengthen the malaria control activities.

**Methods:**

Prevalence of malaria was assessed through a sample blood survey (SBS) in seven randomly selected community health centres (CHCs). Individuals of all age groups in the villages selected (one in each subcentre) were screened for malaria infection. Both thick and thin smears were prepared from blood samples collected by finger prick, stained and examined for malaria parasites searching 100 fields in each smear. The results of a blood survey (n = 10,733) carried out, as a part of another study, during 1986–87 covering a population of 17,722 spread in 37 villages of Koraput district were compared with the current survey results. Software SPSS version 16.0 was used for data analysis.

**Result:**

During the current study, blood survey was done in 135 villages screening 12,045 individuals (16.1% of the total population) and among them, 1,983 (16.5%) were found positive for malaria parasites. *Plasmodium falciparum* was the major malaria parasite species accounted for 89.1% (1,767) of the total positives; *Plasmodium vivax* and *Plasmodium malariae* accounted for 9.3% (184) and 0.2% (5), respectively. Gametocytes were found in 7.7% (n = 152) of the positive cases. The majority of parasite carriers (78.9%) were afebrile. The 1986–87 blood survey showed that of 10,733 people screened, 833 (7.8%) were positive for malaria parasites, 714 (85.7%) with *P*. *falciparum*, 86 (10.3%) with *P*. *vivax*, 12 (1.4%) with *P*. *malariae* and 21 (2.5%) with mixed infections.

**Conclusion:**

The results of the current study indicated a rising trend in transmission of malaria in Koraput district compared to the situation during 1986–87 and indicated the necessity for a focused and reinforced approach for the control of the disease by improving people’s access to diagnosis and treatment and ensuring implementation of the intervention measures with adequate coverage and compliance.

## Background

The goals set by the World Health Assembly and the Roll Back Malaria (RBM) Partnership to reduce the number of malaria cases and deaths recorded in 2000 by 50% or more by the end of 2010, and by 75% or more by 2015 have not yet been achieved [1]. Malaria continues to be one of the most important public health problems with which Odisha State, India is confronted perennially, and there are no signs of it abating [2]. With only 4% of the country’s population, Odisha State contributed 43.9% of *Plasmodium falciparum* malaria and 25.5% of malaria deaths (n = 239) reported in the country during 2008 [3]. Malaria is highly complex in Odisha because of the State’s vast tracts of forest with tribal settlements. The dynamics of malaria vary from place to place [4]. The disease is geographically distributed but remains entrenched in poor population groups particularly in hilly and forested areas characterized by high incidence and deaths due to *P*. *falciparum* infection [5]. Of the 30 districts of Odisha State, Koraput district is one such area with many hills and forests, accounting for 8.5% of all malaria cases, 9.4% of *P*. *falciparum* malaria and 6.3% of deaths due to malaria, during 2008 [3]. Although earlier studies showed that this district is hyperendemic for malaria [6], no scientific studies have been carried out after 1987 on the epidemiology of malaria in this district. This paper summarizes the results of a study on the magnitude of malaria prevalence carried out in seven community health centres (CHC) in Koraput district; the outcome could be useful for the design of appropriate site-specific malaria control strategy.

## Methods

### Study sites and topography

Koraput district has a population of 13,05,492 (2008) spread in an area of 8,807 sq km, and is situated in the southern part of Odisha State (18^0^13’ and 19^0^10’ north latitude and 82^0^5’ and 83^0^13’ east longitude). Some 21% (1,879.53 sq km) of the total area is covered with forest [7]. The sample blood survey was carried out during August and September 2009 in seven CHCs of the district: Dasamantapur, Laxmipur, Nandapur, Kunduli, Pottangi, Lamtaput and Mathalput. The terrain is highly undulating and the villages are located on hilltops or on the slopes of hillocks, criss-crossed by streams.

### Climate

The climate of the district is characterized by hot summer (March–June), rainy (July–October) and cold (November–February) seasons. The monsoon generally breaks during the later part of June each year. Average annual rainfall recorded during 2007 was 2,278.0 mm, of which >80% occurred during July to September under the influence of the south-west monsoon. Maximum rainfall was in August. The mean minimum temperature varied between 14.0°C (January–February) and 27.5°C (April–May) and mean maximum temperature between 27.0°C (December) and 41.5°C (May) [7].

### Demographic features

Of the 61 tribes living in Odisha State, 51 inhabit Koraput district, constituting more than 55% of the district’s total population, distributed in 14 CHCs. The important aboriginal tribes in the district include Porojas, Kondhs, Bondas, Gadabas, Koyas, Bhumiyas, Amanathyas and Gonds. The major occupation of the people is agriculture and collection of forest products. The people of the district, in general, are economically poor, their literacy rate is 35.7% against the State’s 63.1% [7], and they have immense faith in sorcery and witchcraft. The villages are scattered and the houses are built of thatched or tiled roof and mud wall. Houses are generally dark, damp and often without ventilation. Cattle sheds are adjacent to human dwellings and very often domestic animals, such as chicken and goats are also sheltered in the house.

Based on topography, the villages in the district could be broadly classified into three ecotypes: hilltop, foothill and plain. Villages that are situated on hilltops or on slopes are grouped into hilltop ecotype. Perennial streams are the only source of water in these villages. Foothill ecotype includes villages that are located within 0.5 km from foot of hills. Streams, rivulets, terraced paddy fields, wells and ponds are the major breeding habitats in this ecotype. Villages located on flat but undulating land and at least 2 km away from foot of hills are the plain ecotype. Wells, ponds and paddy fields are the major source of Anopheline breeding in this ecotype.

To assess malaria prevalence, among the 14 CHCs of Koraput district, seven CHCs were randomly selected and a sample blood survey was carried out in the CHCs. The total population of the seven CHCs was 5,04,207 comprising 140 subcentres (subdivision of a CHC) and 2,107 villages. All subcentres in each of the seven CHCs were covered for the blood survey by selecting randomly at least one village in each subcentre. Due to logistic problems, five selected villages could not be surveyed. The required sample size for the blood survey with 95% confidence was worked out to be 13,000 assuming an average API of 20 and allowing an error of 12%. The sample size for each CHC was estimated based on proportion to the population.

Informed consent was obtained from the village committee first and then from the persons from who finger-prick blood samples were collected. In the case of children, informed consent was obtained from their parents/caregivers. Ethical clearance was obtained from the ethical committee of Vector Control Research Centre, Puducherry for conducting the blood survey. During the time of blood smear collection, body temperature of fever and non-fever cases was recorded using a clinical thermometer. Afebrile parasitaemia is defined as any parasite load (by microscopy) greater than zero without fever or negative history of fever during the previous 48 hours from the time of taking blood smear. Presumptive radical treatment at appropriate doses of chloroquine and primaquine was administered to all fever cases as per the guidelines of the national programme. Thick and thin smears were prepared on clean slides using the blood samples obtained by finger prick. All blood smears were stained with Giemsa and examined for malaria parasites at 5x100 magnifications searching 100 fields in each thick smear. As a quality control procedure, all positive and 10% of the negative slides were cross-examined following the national guidelines [8].

Using the sample blood survey data, overall parasite prevalence was estimated. The number of parasites in thick smears was graded as 1+, 2+, 3+ and 4+ [9]. The blood smears positive for gametocytes were also recorded. The age-wise break-up of the total as well as the sampled population was done according to Bruce-Chwatt, 1985 [9].

### Data analysis

Data were entered into Microsoft Excel spreadsheet and statistical analyses were carried out using SPSS version 16.0. To compare the malaria parasite prevalence between different categories, *χ*^2^ test was used. To assess risk factors for malaria positivity, logistic regression analysis was carried out. Probability level of P < 0.05 was used for statistical significance.

### Comparison with an earlier study

During September 1986 to July 1987, as a part of another study, a blood survey was conducted covering a population of 17,722 in 37 villages of Koraput district and screening a total of 10,733 persons with a coverage of 60.6%, and the results have been discussed elsewhere [10]. The salient features of those results on malaria prevalence are recapitulated and presented in the Results section of the current paper, and compared with the corresponding data obtained during the present study.

## Results

**Figure 1:**
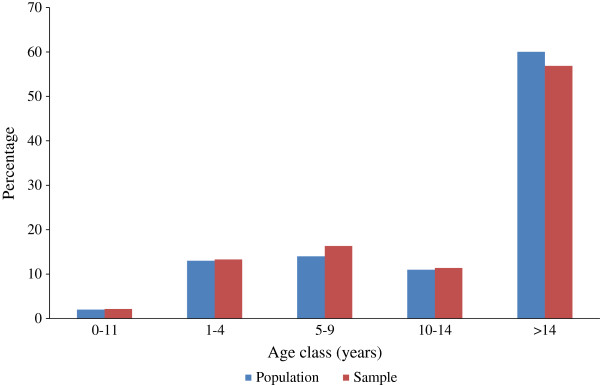
Age-specific distribution of the total and the sampled population.

The study population was 74,944, distributed in 135 villages (Table 1). In total, 12,045 (16.1%) blood smears were collected and the proportion of population covered varied between 13.6% in Laxmipur CHC and 20.8% in Pottangi CHC. Age-specific distribution of the total and the sampled population is shown in Figure 1. There was no significant variation (P > 0.05) in the age-specific distribution pattern of the total and the sampled population, indicating an appropriate representation of all age classes among the sampled population. Across different age classes, the percent sampled for blood examination varied from 15.3 among >14 year olds to 18.6 among five to nine year olds (Table 2). Out of 12,045 blood smears examined, 1,983 (16.5%; 95% confidence limits: 15.8%-17.1%) were found positive for malaria parasites. The slide positivity rate (SPR) in the seven CHCs varied from 5.7% in Nandapur to 40.6% in Laxmipur (Table 3). There was a significant variation in the slide positivity rates between the CHCs (*χ*^2^ = 1,243; P < 0.001). Overall, SPR among males (18.1%) was significantly (*χ*^2^ = 22.6; P < 0.001) higher than that of females (14.9%) (Table 3). For further analyses to study the epidemiology of malaria situation, data from all seven CHCs were combined and the results are presented. 

**Table 1:** Number of villages and population covered by the sample blood survey in Koraput district

**Sl No**	**CHC**	**Population**	**Number of subcentres**	**Number of villages**	**Villages selected**	**Total population in the selected villages**	**% covered**
1	Mathalput	71525	21	230	21	12540	14.9
2	Dasamantapur	69803	20	290	19	11364	15.5
3	Lamtaput	56224	18	248	18	8886	15.6
4	Kunduli	81259	22	254	21	12384	14.1
5	Laxmipur	61772	16	176	16	11723	13.6
6	Nandapur	94852	26	482	24	10456	20.2
7	Pottangi	68772	17	427	16	7591	20.8
Total		504207	140	2107	135	74944	16.1

CHC = community health centre.

**Table 2:** Slide positivity rate (SPR) among different age groups

**Sl No**	**Age groups**	**Population**	**BSE**	**% covered**	**Positive**	**SPR (%)**
1	0-11 months	1499	260	16.9	47	18.1
2	1-4 years	9742	1602	16.4	348	21.7
3	5-9 years	10493	1965	18.6	367	18.7
4	10-14 years	8243	1372	16.7	246	17.9
5	> 14 years	44967	6846	15.3	975	14.2
Total		74944	12045	16.1	1983	16.5

**Table 3:** Gender-specific slide positivity rate in different community health centres (CHCs)

**CHC**	**Females sampled**	**Positivity rate (%)**	**Males sampled**	**Positivity rate (%)**	**Total sampled**	**Positivity rate (%)**	**Comparison of rates between gender**	
							**Chi-square**	**P-value**
Dasamantapur	888	26.13	870	29.43	1758	27.76	2.38	0.122
Kunduli	845	7.10	906	9.05	1751	8.11	2.23	0.135
Lamtaput	755	7.68	627	11.16	1382	9.26	4.94	0.026
Laxmipur	750	40.40	844	40.76	1594	40.59	0.02	0.884
Mathalput	1016	7.28	849	10.37	1865	8.69	5.54	0.019
Nandapur	1080	4.81	1036	6.66	2116	5.72	3.34	0.068
Pottangi	854	16.74	725	20.97	1579	18.68	4.60	0.032
Total	6188	14.90	5857	18.12	12045	16.46	22.6	0.000

Malaria SPRs, with 95% confidence interval, across the three ecotypes, hilltop, foothill and plain villages were 27.63% (30.4-24.85%), 17.88% (18.79-16.97%) and 11.57% (12.53-10.61%), respectively. There was a significant (*χ*^2^ = 174.5; P = 0.000) variation in SPRs between the three ecotypes. However, there was no significant (*χ*^2^ = 14.82; df = 8; P = 0.063) variation in malaria parasite species distribution between the ecotypes.

Logistic regression analysis was carried out by considering SPR (0-negative, 1-positive) as dependent variable and the other study variables, such as age, gender and ecotype, as independent variables. The overall fit of the logistic model was found to be good fit (Hosmer and Lemeshow Test: *χ*^2^ = 12.68; df = 8; P = 0.123). Individual co-efficient of independent variables was also found to be statistically significant (P < 0.05). The estimated odds ratio (OR) with 95% confidence interval for females was 0.84 (0.76-0.93) indicating that the risk of getting malaria among females is significantly (P < 0.001) less than that of males (OR = 1.0). Similarly the OR for foothill (0.58; 95% CI = 0.5-0.67) and plain areas (0.35; 95% CI = 0.29-0.41) were significantly (P < 0.001) less than that of hilltop villages (OR = 1).

Age-specific SPR is presented in Table 2. The SPR was observed to be age dependent (*χ*^2^ trend in proportion: 60.52; P < 0.005). Three human malaria parasite species were detected during the study and *P*. *falciparum* was the predominant one accounting for 89.1% (1,767) of the total infections, *Plasmodium vivax* and *Plasmodium malariae* accounted for 9.3% (184) and 0.2% (5), respectively. Mixed infection of more than one species was found in 27 (1.4% of all infections) infected individuals. Mixed *P*. *falciparum* and *P*. *vivax* infection was the most common (92.6%), followed by *P*. *falciparum* and *P*. *malariae* (7.4%) (Table 4). 

**Table 4:** Species composition of malaria parasites among different age groups

**Sl No**	**Age group**	**Total positives**	***Pf***	***Pv***	***Pm***	***Pf *and *Pv***	***Pf *and *Pm***	***Pv *and *Pm***
1	0-11 months	47	39	7	0	1	0	0
2	1-4 years	348	294	42	0	11	1	0
3	5-9 years	367	325	38	1	2	1	0
4	10-14 years	246	216	26	1	3	0	0
5	Above 14 years	975	893	71	3	8	0	0
Total		1983	1767	184	5	25	2	0

*Pf* = *Plasmodium falciparum*; *Pv* = *Plasmodium vivax*; *Pm* = *Plasmodium malariae*.

In total, 152 (7.7%) malaria positive cases had gametocytes in their blood. Among the *P*. *falciparum*, *P*. *vivax* and *P*. *malariae* positive cases, 7.0% (123), 14.7% (27) and 40% (two) had gametocytes, respectively. Among the total *P*. *falciparum* cases (1,767), the highest gametocyte rate of 8.6% (25 out of 292) was found among one to four years age group followed by 8.2% (27 out of 330) among five to nine years. The gametocyte rate among 10–14 and >14 years was 3.7% and 6.8%, respectively. Among the infants, the gametocyte rate was 5.1% (two out of 39). However, there was no evidence of age dependence (*χ*^2^ = 0.214; P = 0.6434) on gametocyte rate. Similarly, among the total *P*. *vivax* infections (184), 14.1% was with gametocytes. The highest gametocyte rate of 28.6% (two out of seven) was recorded among infants followed by 23.7% (nine out of 38) among five to nine years. There was no significant difference (P > 0.05) in gametocyte rates between different age groups.

Age-wise distribution of infected individuals by parasite density is given in Table 5. In all age classes, more than 75% of the infected individuals had density of 2+ or less. In *P*. *falciparum* and *P*. *vivax* cases, 1+ density was the highest followed by 2+, 3+ and 4+ (Table 6). There was no significant difference (*χ*^2^ = 2.05; P > 0.05) in the parasite density pattern between *P*. *falciparum* and *P*. *vivax*. 

**Table 5:** Distribution of malaria positives by parasite counts among different age groups

**Sl No**	**Age groups (in years)**	**Total positives**	**Number of positive cases with**			
			**+ (%)**	**++ (%)**	**+++ (%)**	**++++ (%)**
1	<1	47	26 (55.3)	7 (14.9)	10 (21.3)	4 (8.5)
2	1-4	348	179 (51.4)	102 (29.3)	43 (12.4)	24 (6.9)
3	5-9	367	175 (47.7)	105 (28.6)	63 (17.2)	24 (6.5)
4	10-14	246	121 (49.2)	72 (29.3)	33 (13.4)	20 (8.1)
5	> 14	975	523 (53.6)	302 (31.0)	103 (10.6)	47 (4.8)
Total		1983	1024 (51.6)	588 (29.7)	252 (12.7)	119 (6.0)

**Table 6:** Malaria positives among male and female populations by parasite count

**Species**	**Density**	**Females**	**Males**	**Total**	P-values*
*Pf*	+	430 (52.6)	494 (52.1)	924 (52.3)	0.2933
	++	231 (28.2)	284 (29.9)	515 (29.1)	0.1505
	+++	105 (12.8)	114 (12.0)	219 (12.4)	0.0483
	++++	52 (6.4)	57 (6.0)	109 (6.2)	0.0283
	Total	818	949	1767	
*Pv*	+	52 (57.1)	45 (48.9)	98 (53.0)	0.0605
	++	28 (30.8)	30 (32.6)	58 (31.7)	0.8400
	+++	8 (8.8)	13 (14.1)	21 (11.5)	0.0151
	++++	3 (3.3)	4 (4.3)	7 (3.8)	0.3417
	Total	91	92	183	
*Pm*	++	0	5	5	

Figures in parenthesis are the percentage of different parasite counts of the total malaria positive cases by gender.

*Pf* = *Plasmodium falciparum*; *Pv* = *Plasmodium vivax*; *Pm* = *Plasmodium malariae*

*Based on Chi-square test.

Afebrile parasitaemia was confirmed in 78.9% of the cases and the remainder (21.1%) were febrile. There was no significant difference (P > 0.05) in proportion of afebrile cases with the three parasite species (78.3% *P*. *falciparum*, 77.7% *P*. *vivax* and 80% *P*. *malariae* infections). However, in the case of mixed infections, the trend was in reverse order, 40.7% was afebrile and 59.3% was febrile. Age-wise analysis of malaria positives showed that afebrile cases ranged from 73.2% to 81.1% among the five age groups (under 11 months, one to four, five to nine, 10–14 and >14 years) and there was no significant (*χ*^2^ = 4.64; P = 0.3264) difference between them (Table 7). 

**Table 7:** Age-wise afebrile and febrile malaria positive cases

**Sl No**	**Age groups**	**Number of positives**	**Number of cases**	
			**Afebrile (%)**	**Febrile (%)**
1	0-11 months	47	36 (76.6)	11 (23.4)
2	1-4 years	348	272 (78.2)	76 (21.8)
3	5-9 years	367	286 (77.9)	81 (22.1)
4	10-14 years	246	180 (73.2)	66 (26.8)
5	Above 14 years	975	791 (81.1)	184 (18.9)
Total		1983	1565 (78.9)	418 (21.1)

**Figure 2:**
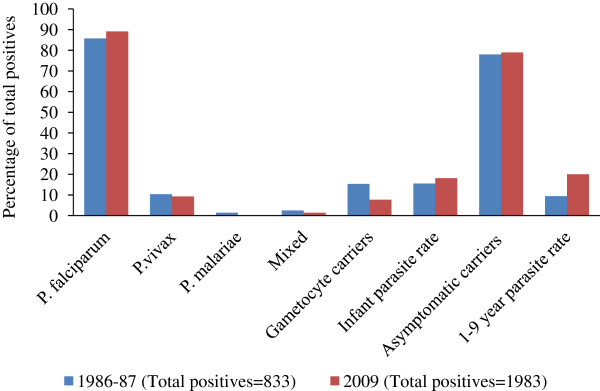
Comparison of parasite species composition and other parameters between 1986–87 and 2009 surveys.

The data on malaria prevalence and other parameters obtained from the survey conducted during 1986–87 in Koraput district were compared with the data collected during the current survey (Figure 2). The malaria prevalence (SPR) presently recorded in the district was significantly (*χ*^2^ = 395.4; P < 0.001) higher (16.5%) than that (7.8%) reported 22 years ago, i.e., during 1986–87, increased from 7.8% to 16.5%. The proportion of *P*. *falciparum* and *P*. *vivax* remained almost same without variation. However, the prevalence of *P*. *malariae* reduced from 1.4% to 0.2%. Infant parasite rate increased from 15.5% to 18.1%. The prevalence of afebrile carriers in the community remained almost at the same level (78.0% in 1986–87 and 78.9% in 2009). Parasite rate among one to nine years was higher (20.0%) in the 2009 survey compared to the survey in 1986–87 (9.4%). However, the gametocyte rate in the community was found higher in the earlier survey (15.3%) compared to the present survey (7.7%) (Figure 2). 

## Discussion

An evaluation of local problems and an appropriate epidemiologic information system are prerequisites for any malaria control programme. To understand the malaria situation in an area, malaria prevalence is one of the important epidemiological parameters, which can be measured either by mass blood survey or sample blood survey in the area. The earlier blood survey conducted 22 years ago highlighted the prevalence of malaria with hyperendemicity in Koraput district [10]. Subsequently, there was no scientific study on malaria prevalence carried out in the district, although incidence of malaria has been reported by the District Health Department every year from 1988 to 2008; the annual parasite incidence (API) ranged from 10.1 (1992) to 25.1 (2008) [11]. The results of the current survey conducted in 2009 in the same area reconfirmed the hyperendemic situation of malaria and revealed that malaria has been persistent among tribes inhabiting Koraput district. The parasite prevalence that ranged between 5.7% (Nandapur CHC) and 40.6% (Laxmipur CHC) with an average of 16.5% was higher than that (7.8%) recorded in the district during the early 1990s and placed the district in the hyperendemic category [10,12]. The prevalence of malaria was higher in Laxmipur and Dasamantapur CHCs compared to the other five CHCs. This could be due to the fact that most of the villages (>80%) in these two CHCs are situated on hills, which is the highly favourable ecotype for *Anopheles fluviatilis*, the major malaria vector in the area [10,13,14], while in the other five CHCs, less than 50% of the villages are hilly [7]. The publications [6,10] arising from the 1986–87 survey confirmed the lower transmission intensity in foothill and plain villages, as observed in the current study. This could be due to the low prevalence of *An*. *fluviatilis* in foothill and plain villages compared to the hilltops [15]. The significantly (P < 0.05) higher malaria positivity rate recorded in males compared to that in females could be due to the dressing pattern of females who fully cover their bodies and sleep indoors. Males are mostly involved in forest-related activities during evening hours and they are likely to get more mosquito bites, as a result of which the chances of man-vector contact is high in males compared to females. Most of the point prevalence studies in India were carried out for outbreak/ epidemic investigations. There is very limited information on gender-specific malaria prevalence in different paradigms in the country. In the available studies, age and gender classification used is arbitrary [16-22]. The burden of malaria was generally higher in men than women in all age groups. In a recent epidemiological study in Sonitpur district of Assam, it was shown that overall; malaria prevalence (SPR) was higher among males (43.2%) than females (34.5%) [23].

Out of the four human malaria parasites reported earlier in the district [24], three parasite species were recorded in the current study. *Plasmodium falciparum* was the predominant malaria parasite known to be prevalent in the same proportion in Koraput district, even in early part of 19^th^ Century [10,12]. Similar to the findings of the present study, in Sundargarh district of Odisha State, *P*. *falciparum* accounted for 85.0% of the total malaria cases recorded from January 2001 to December 2003 [25]. Mixed infection of more than one parasite species was detected in the current study indicating intense transmission of malaria in the district.

Age-specific analysis indicated that children (one to nine years) had higher prevalence of malaria compared to adults. Similar observation was made in Assam [16,19,20], Arunachal Pradesh [21] and Rajasthan [17], whereas in the Indo-Gangatic plains, the situation was reversed [18,22]. The high prevalence of afebrile parasite carriers (78.9%) among the community indicated that the population had high acquired immunity. Similar observation was also made in the earlier study conducted in this district [10]. Asymptomatic parasitaemia is known to be high in an immune population [6,10,26,27]. In the present study, the same level of asymptomatic parasitaemia was observed in all age groups indicating a high immunity of the population at all age groups and that the study area is highly endemic for malaria.

In the present study, one of the interesting aspects of *P*. *falciparum* infection was the prevalence of low number of gametocyte carriers (7.0%) though a large proportion of population had the infection. This could be due to the consequence of launching Enhanced Malaria Control Project (EMCP) in India in April 1997 covering 100 districts (including Koraput district) and 19 urban areas in eight peninsular states [28]. Under the EMCP, all suspected/clinical malaria cases were treated with presumptive radical treatment (PRT) (600 mg chloroquine and 45 mg primaquine adult dose) in high risk areas [28]. As most of the villages in Koraput district were coming under the high risk category, primaquine (gametocidal drug for *P*. *falciparum*) was administered to all suspected/ clinical malaria cases and this could be the reason for the low prevalence of *P*. *falciparum* infection with gametocytes. The proportion of overall gametocyte carriers (of all parasite species) was also higher (15.3%) in the earlier survey [10] than that recorded in the present survey (7.7%).

There is no doubt that malaria continues to be a major public health problem causing morbidity and mortality among the tribes in the district and several factors are responsible for the persistence of the disease. As most of the villages in the district recorded API well above two during the last two decades, indoor residual spraying (IRS) of insecticide had been the mainstay of vector control by the malaria control programme in the country. The villages of the district have been receiving yearly two rounds of IRS with DDT at a dosage of 1 g/sq m till date [13]. Active fever surveillance was carried out in the villages at fortnightly interval by the health workers of the CHCs to screen malaria positive cases and treat them with chloroquine. In addition, drug distribution system was established by opening fever treatment depots (FTDs) and drug distribution centres (DDCs) with the help of voluntary agencies [28]. Under the EMCP, besides surveillance and treatment including PRT, around 24,000 insecticide treated mosquito nets (ITNs) were distributed in one of the CHCs in the district covering a population of 65,000 [14].

However, since the villages in the district are scattered and most of them are located on hilltops or slopes of hillocks, communication facilities and health care services were poor and inadequate and often led to breakdown of regular malaria control operations. Insecticide spraying operations could not be carried out in time due to difficult terrain and inaccessibility of many villages. Even if sprayed, the coverage and quality of spraying were poor due to lack of supervision. More than 55% of the houses could not be sprayed due to refusal [29]. Moreover, the sprayed surfaces of the houses are mud-plastered by the villagers within 3 days after spraying. Thus, it was found that indoor residual spraying of insecticide was less acceptable to the community [29]. Further, the benefits of ITNs could not be sustained due to the retreatment problem [14]. Hence, from 2010, the malaria control operation in the entire Odisha State has gradually been shifted towards reducing areas under IRS and increasing the coverage with long lasting insecticidal nets (LLINs) that are expected to be effective for more than three years [30] to make a discernible impact. Case detection and access to treatment were insufficient, as the health workers devoted more time to perform other activities of the CHCs. On the other hand, the tribal people exhibited poor health seeking attitude due to their low literacy rate (35.7%), disbeliefs and misconceptions. They generally adopt their native remedies and witchcraft, as chloroquine was not readily available to them always. In recent past, chloroquine-resistant *P*. *falciparum* strain was detected with increasing frequency in many districts of Odisha State as well as in the country [31]. Hence, the national malaria drug policy in the country was revised in 2010 and in the revised policy it was recommended that the first line of treatment of all uncomplicated *P*. *falciparum* cases in the entire country will be artemisinin-based combination therapy (ACT) [31]. The introduction of ACT and rapid diagnostic kits (RDKs) in Koraput district and delivery of drugs through accredited social health activists (ASHAs) (trained female community health activist selected from the village itself) are expected to enhance people’s access to diagnosis and treatment of malaria. The prevailing malaria situation needs a determined effort to implement the newly introduced intervention tools such as LLIN and ACT in order to curtail the transmission of malaria in the district.

## Competing interests

The authors declare that they have no competing interests.

## Authors’ contributions

SSS and KG conceived and designed and performed the study. PV compiled and analysed the data. SSS and KG drafted the manuscript. PJ critically reviewed the manuscript. All authors contributed to the writing of the manuscript and approved the final version.
